# Supplementary material for: Ni-Catalyzed Enantioselective Intramolecular Mizoroki–Heck Reaction for the Synthesis of Phenanthridinone Derivatives
Source: J Org Chem. 2023 Jun 15;88(13):8203–26. doi: 10.1021/acs.joc.3c00202 (PMC10337041; doi:10.1021/acs.joc.3c00202)

YK 94-078 check CDCl3 H
PROTON CDCl3 {D:\nmrusers\malachowski} BM 16

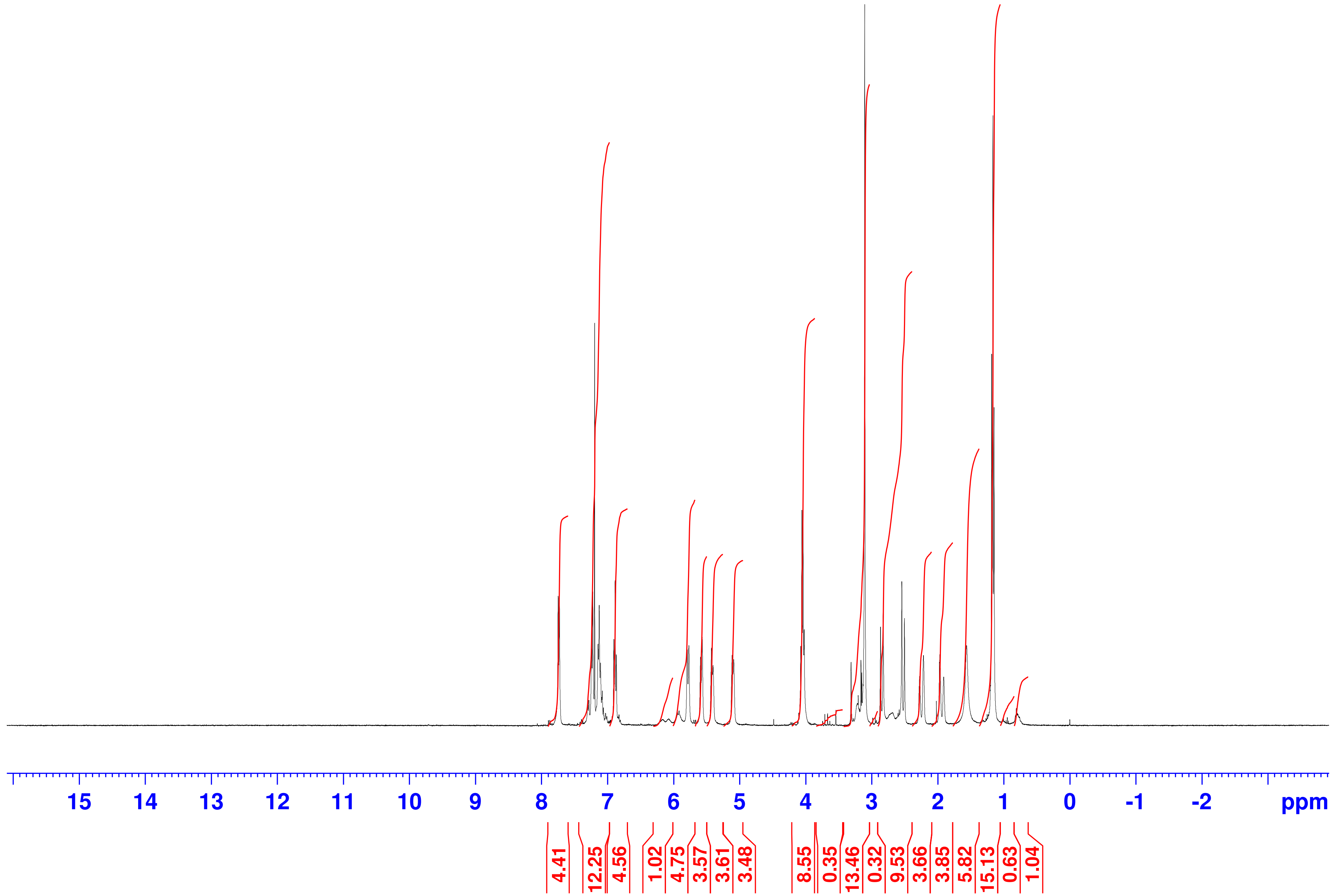

Supplement: Supplementary file 2 — jo3c00202_si_002.zip [file jo3c00202_si_002.zip › Amides(N-H and N-Me)/1g-I EtCO2CH2-NMe-ArI/1H EtCO2CH2-NMe-ArI 1g-I/5/pdata/1/email_YK 94-078 check CDCl3 H_5_1.pdf]
